# Assessing Static Balance, Balance Confidence, and Fall Rate in Patients with Heart Failure and Preserved Ejection Fraction: A Comprehensive Analysis

**DOI:** 10.3390/s24196423

**Published:** 2024-10-04

**Authors:** Andriana Teloudi, Maria Anifanti, Konstantinos Chatzinikolaou, George Grouios, Vassilia Hatzitaki, Ioanna Chouvarda, Evangelia Kouidi

**Affiliations:** 1 Laboratory Sports Medicine, Department of Physical Education & Sport Science, Aristotle University of Thessaloniki, 57001 Thessaloniki, Greece; teloudia@phed.auth.gr (A.T.); manyfant@phed.auth.gr (M.A.); 2 Laboratory of Motor Behavior and Adapted Physical Activity, Department of Physical Education & Sport Science, Aristotle University of Thessaloniki, 57001 Thessaloniki, Greece; konchatzinikolaou@phed.auth.gr (K.C.); ggrouios@phed.auth.gr (G.G.); vaso1@phed.auth.gr (V.H.); 3 Laboratory of Computing, Medical Informatics and Biomedical-Imaging Technologies, School of Medicine, Aristotle University of Thessaloniki, 54124 Thessaloniki, Greece; ioannach@auth.gr

**Keywords:** heart failure with preserved ejection fraction, static balance, posture control, falls, balance confidence, Center of Pressure, bipedal stance, Tandem-Romberg stance

## Abstract

Chronic heart failure (CHF) is a complex clinical syndrome, associated with frailty, higher fall rates, and frequent hospitalizations. Heart Failure (HF) and preserved ejection fraction (HFpEF) is defined as a condition where a patient with HF have a diagnosis of left ventricular ejection fraction (LVEF) of ≥ 50%. The risk of HFpEF increases with age and is related to higher non-cardiovascular mortality. The aim of this study was to evaluate static balance and examine the effect of task difficulty on the discriminating power of balance control between patients with HFpEF (Patients with HFpEF) and their healthy controls. Moreover, the associations between static balance parameters, balance confidence, falls, lean muscle mass, and strength were assessed. Seventy two patients with HFpEF (mean age: 66.0 ± 11.6 years) and seventy two age- and gender-matched healthy individuals (mean age: 65.3 ± 9.5 years) participated in this study. Participants underwent a 30 s bilateral stance (BS) test and a 20 s Tandem-Romberg stance (TRS) on a force platform, evaluating the Range and Standard Deviation of Center of Pressure (COP) displacement parameters in both axes. Balance confidence was evaluated by the Activities-Specific Balance Confidence (ABC) Scale, and the number of falls during the last year was recorded. Lower limb strength was measured using an isokinetic dynamometer, isometric leg strength, and a Sit-to-Stand test. Bioelectrical impedance analysis was conducted to assess lean fat mass, lean fat mass index, and lean%. Patients with HFpEF presented with lower static balance in BS and TRS compared to healthy controls (*p* < 0.05), lower balance confidence by 21.5% (*p* < 0.05), and a higher incidence of falls by 72.9% (*p* < 0.05). BS was a better descriptor of the between-group difference. Furthermore, static balance, assessed in controlled lab conditions, was found to have little if no relationship to falls, strength, lean muscle mass, and balance confidence. Although no correlation was noted between the static balance parameters and falls, the fall rate was related to balance confidence, age, muscle strength, and lean fat.

## 1. Introduction

Heart Failure (HF) is a complex, progressing, clinical syndrome related to different etiologies and pathophysiology [1]. It is characterized by symptoms, such as dyspnea, fatigue, swelling, and/or decreased exercise capacity, which are associated with structural and/or functional cardiac abnormalities, resulting in elevated intracardiac pressures or reduced cardiac output [1,2,3].

Patients with HF and preserved ejection fraction (HFpEF) have the diagnosis of HF with left ventricular ejection fraction (LVEF) of 50% or higher [4]. Of the 1–2% of people living with HF worldwide, it is estimated that more than half of them are patients with HFpEF [5,6]. Despite having similar outcomes regarding hospital admissions to patients with HF and reduced ejection fraction [7], individuals with HFpEF have better survival rates [8]. Nevertheless, deaths from non-cardiovascular causes are greater in patients with HFpEF [6,9,10].

Among other characteristics, patients with HFpEF experience dyspnea and exercise intolerance that impair their performance in aerobic activities (e.g., 6 MWT) or exercises that require strength [11,12]. Cardiac and peripheral abnormalities, including skeletal muscle and vascular dysfunction, are associated with reduced strength and exercise capacity in patients with HFpEF [12,13,14]. Furthermore, mitochondrial disorders at the cellular level contribute to skeletal muscle metabolic abnormalities in patients with HFpEF [12]. The reduced total and leg lean muscle mass and the incrementation of adipose tissue may have a negative impact on mitochondrial density and biogenesis, resulting in decreased exercise tolerance and reduced muscle strength [15,16]. 

In patients with cardiovascular diseases, muscle mass and muscle strength are reduced with advancing age, considering that skeletal muscle may be replaced by non-functional mass, such as fat tissue, without any body mass index (BMI) changes [17]. This phenomenon is linked to lower quality of life and frailty syndrome, considering that sarcopenia is an independent predictor of death [17,18]. Among patients with HFpEF, a reduced lean/fat ratio is linked to a reduced exercise tolerance, cardiovascular health deterioration, an increase in hospitalization admissions, and mortality [19]. Nevertheless, lean body mass incline among patients with HF is related to lower 1-year mortality rates [20]. 

The development or maintenance of skeletal muscle strength is crucial for producing human movement, and thereby facilitating the execution of daily activities [21]. Due to reduced muscle mass and strength, HF patients often encounter adverse events, including falls, hospitalization, and comorbidities, affecting their balance, functional independence, fear of falling, and quality of life [22,23,24]. It is estimated that more than 60% of hospitalized cardiac patients are moderately to highly prone to falls [25], causing severe physical and economic costs to themselves and increasing the burden on the health system [26]. There are various reasons for falls, including multiple medications, syncope or autonomic nervous system dysfunction, frailty matters, sensory impairments, cognitive problems, or postural control issues [27]. According to Denfeld et al. [28] individuals with HFpEF and a greater body mass index are more susceptible to falling. 

Recent studies demonstrated a significant relationship between free fat mass and fall rates in frail older adults [29]. Patients with HFpEF exhibited significantly lower percentages of total lean body and leg mass compared to age-matched healthy controls, indicating the existence of skeletal muscle atrophy [30]. Loss of muscular strength, mass, and/or function, detected as sarcopenia, was 20% higher in patients with HF than in age-matched healthy controls [31,32]. This phenomenon was linked to faster progression of cardiovascular diseases, as well as an increased risk of death, falls, and a lower quality of life, particularly in older people [33]. Furthermore, one in four older individuals mention a fall each year [34], and older people with HF encounter 14% greater odds of having a fall compared to healthy controls [35]. Notwithstanding, there is a lack of studies examining the relationship between lean muscle mass, falls, and static balance in patients with HFpEF.

Skeletal muscle fatiguability is often higher in patients with HF, especially in the lower limbs [36]. Lower leg strength was associated with a poor prognosis, related to reduced exercise tolerance, physical inactivity, and health status [37]. Ankle dorsiflexion muscle strength was discovered to be an independent predictor for increased fall risk in KTRs [38]. The STS test was identified as a useful tool for detecting the incidence of falls [39]. Particularly, STS leg muscle power was regarded as a more predictive factor of everyday living falls than isometric strength in community-dwelling older adults [40]. Another study supported that hip abductor strength and hip flexor power are related to prospective falls in community-dwelling older adults [41]. Tanriverdi et al. [42] reported higher rates of falls among patients with HF, claiming that a greater number of falls may be related to ejection fraction, functional exercise capacity, and balance. Nevertheless, a question remains, whether there is a correlation between muscle strength and falls in patients with HFpEF.

Balance is described as the result of constant communication between the central and peripheral nervous systems for maintaining the body’s center of gravity within the base support [43]. Deficits in postural control, in sarcopenic older adults, are related to augmented risk and fear of falling [24]. Patients with HF show impaired static and dynamic (i.e., during gait) balance control compared to healthy age-matched controls [42,44]. In most cases, however, balance is assessed with clinical scales and field tests, which do not allow a thorough consideration of the underlying control impairments [44,45]. In the few studies assessing balance in the laboratory using a force platform, the one-leg stance test was used, considering this test is a predictor of injurious falls among the elderly [42,46]. Moreover, previous studies examining balance in HF patients included participants with reduced LVEF and had small sample sizes. In addition to bipedal stance, the Tandem-Romberg test is a valid and simple static balance task that challenges balance to a greater extent, improving the chance to recognize older adults who are susceptible to falls [47]. How static balance tasks of increasing difficulty can discriminate patients with HFpEF from healthy controls is not known yet. Furthermore, whether performance in classical laboratory static balance tests is associated with indices of muscle mass and strength, as well as fall incidence in patients with HFpEF is an unexplored issue. 

Balance confidence, measured with the ABC scale claimed to be a significant predictor of falls in the elderly, indicating that a low test score is positively associated with a higher likelihood of falling [48]. Similarly, it is reported that elderly individuals who experience falls present lower balance confidence and increased fear of falling than non-fallers [24,44,49] demonstrating that patients with HF who had low ABC scores were at high risk of falling. According to Myers et al. [50], ABC scores above 50 and below 80 indicate a moderate degree of dysfunction.

Considering the above, our recent study focuses on examining static balance, balance confidence, and the frequency of falls and their relationship using field tests in combination with up-to-date laboratory procedures and force plates. According to the study design, there was an attempt to identify the aspects related to static balance and factors responsible for falls in patients with HFpEF. Thereafter, our results were discussed in comparison with the findings of recent publications. Finally, new approaches were presented for assessing static balance, balance confidence, and falls in patients with HFpEF.

The purpose of this study was to evaluate static balance, discriminating differences within the two static balance tasks (bipedal and Tandem-Romberg) in patients with HFpEF compared to healthy controls. A secondary aim was to assess the associations between static balance parameters, balance confidence, falls, lean muscle mass, and strength. 

## 2. Materials and Methods

### 2.1. Study Population

Following an open invitation from the Laboratory of Sports Medicine of Aristotle University of Thessaloniki, Greece, individuals who were interested in participating in this study contacted the Laboratory of Sports Medicine and were screened to verify if they met the eligibility criteria. Individuals aged over 18 years, who: (a) had a confirmed diagnosis of HFpEF, determined by an LVEF ≥ 50%, and showing evidence of impaired diastolic function [51], (b) were clinically stable with typical HF signs and symptoms consistent with New York Heart Association (NYHA) functional class I-III, and (c) voluntarily agreed to participate in the study, were eligible. Exclusion criteria were the presence of the following: (a) acute coronary syndrome (<4 weeks), (b) severe valvular diseases, complex arrhythmias, poor regulation of comorbidities, (c) mental or cognitive impairment, (d) vestibular disorders, (e) history of orthopedic and/or neurologic problems, limiting mobility, (f) concurrently engaged in a structured exercise training program. Moreover, age- and sex-matched healthy volunteers, with no history of chronic diseases, or orthopedic/musculoskeletal problems, who volunteered, were eligible to participate in this study.

After examining the medical records of the volunteers from both groups, all eligible individuals received comprehensive details regarding the study protocol and provided written informed consent. The study protocol was evaluated and approved by the Ethics Committee of the Department of Physical Education and Sports Science of Aristotle University of Thessaloniki (Protocol number: 110/2022). Furthermore, the clinical trial was registered on ClinicalTrials.gov (NCT06036615).

### 2.2. Study Design

Volunteers who fulfilled the inclusion criteria and voluntarily participated undertook the assessment, which included the participant’s demographic variables, medical history, clinical examination (blood pressure measurement, ECG, echocardiogram), body composition analysis, evaluation of static balance on a force plate, 30 s Sit-to-Stand test, and leg strength test. Activities-Specific Balance Confidence (ABC) Scale and number of falls were recorded. All functional capacity measurements were conducted in the morning, between 9.00 a.m. and 13.00 a.m., with an adequate rest period between the tests, by the same researchers who were blinded to the group allocation. Participants were informed to refrain from eating for at least four hours and to abstain from caffeine (i.e., normal tea, coffee, and energy drinks), alcohol consumption, and any form of exercise for at least 12 h.

### 2.3. Clinical Examination and Echocardiography

The evaluation included a detailed medical history (etiology of HF, hospitalizations, medication, comorbidities) and a resting 12-lead electrocardiogram (ECG) (Mac 600, GE Medical, 8200 W, Tower Avenue, Milwaukee, WI, USA). Echocardiographic studies were performed in all participants using Vivid S70 (GE Medical; Horten, Norway) equipped with an M5 S phased-array transducer. All images were stored (EchoPAC, version 204) and then analyzed by two cardiologists, according to the European Association of Cardiovascular Imaging and American Society of Echocardiography guidelines [52,53]. LVEF was calculated using the modified biplane Simpson’s method. The diastolic assessment was estimated by the measurements of early diastolic transmitral flow velocity (E), late diastolic transmitral flow velocity (A), and their E/A ratio. Pulsed-wave TDI analysis was performed to measure the early diastolic mitral annular velocity (E’) from the lateral and septal walls. The average E’ (E’ av) was calculated from both walls and the ratio E/E’av was measured as an index of LV filling pressures.

### 2.4. Body Composition Analysis

Dry weight (kg) and height (cm) were measured using a SECA electronic scale with a stadiometer (model 220, Hamburg, Germany). The bioelectrical impedance analysis (BIA) was performed using a QuadScan 4000 Touch (Bodystat, Warwickshire, UK). Participants were placed in a supine position and two pairs of disposable electrodes were positioned on the foot (under the base of the toes and on the ankle, between the medial and lateral malleoli) and the hand (below the dorsum of the hand, over the third metacarpophalangeal joint and the wrist). Lean%, lean fat mass (kg), body free fat mass index (FFMI), and body mass index (BMI) were assessed. Lean fat mass depicts the lean muscle mass where muscles, bones, and water are included. BMI was calculated using the equation BMI = Weight/Ht^2^ in metric form, and FFMI using the equation FFMI = Lean/Ht^2^ in metric form. 

### 2.5. Evaluation of Static Balance

Prior to testing, participants were introduced to the laboratory premises and given detailed information regarding the procedure. The procedure was firstly explained verbally, followed by a demonstration of each task and a description of safety measures that were implemented, in case of necessity (i.e., a chair next to the patient). Thereafter, the participants were familiarized with the procedure on the wooden floor barefooted and then on the force plate, for approximately 1–2 min. Participants were encouraged to stay focused and stand as still as possible. 

For the assessment of static balance, the Bertec force plate (dimensions of 60 cm × 40 cm, model 6501, BERTEC Corporation, Columbus, OH, USA, sampling rate 960 Hz) was used. The force plate recorded the vertical ground reaction force and the two moments of force about the anteroposterior (Y) and mediolateral (X) axis, from which the Center of Pressure (COP) displacement along the two axes (COPX, COPY) were calculated. The COP depicts the location of the application of the ground reaction forces under the feet. The platform was synchronized with the VICON motion capture system, and data management was performed using the VICON NEXUS 2.16.0 software. 

Static balance was assessed during the execution of two progressively challenging exercises, in two trials: (a) standing in a bipedal position (feet parallel, with an internal malleolus distance of 10 cm) for a duration of 30 s, (b) Tandem-Romberg stance (placing one foot behind the other so that the toe of one foot touches the heel of the other) for a duration of 20 s. The non-dominant foot was positioned in front of the dominant one for this test, in all participants. The two tasks were performed in a random order. A 30 s rest period was given between the trials. Trials lasting less than 20 s in the Tandem-Romberg stance were not taken into consideration.

Data from each recording were stored on a desktop workstation for further processing. In the VICON NEXUS 2.16.0 software environment, the COPX and COPY timeseries were selected for each trial and downloaded from the first to the last frame in text files. Timeseries were analyzed using custom-built algorithms implemented in MATLAB (v. 2022 b). To reduce the signal-to-noise ratio, a fourth-order, low-pass Butterworth digital filter with a cutoff frequency of 6 Hz was applied to the entire time series of each recording. The participant’s ability to maintain postural stability during each task was assessed by computing the following COP parameters: (a) Range (peak to peak) of COP displacement in the ML and AP directions, (b) Standard Deviation (SD) of the COP displacement in ML and AP directions. To explain the assessment indicators further, the Range depicted the maximum distance between the COP and its reference point, while the SD represented the extent of COP position fluctuation over a period of time.

### 2.6. Lower Limb Strength 

The Baseline leg dynamometer (White Plains, New York, NY, USA) was utilized to assess lower limb strength. The participant was instructed to place his/her feet on the designated space on the platform, bend the knees to semi-squat, and hold the handle of the dynamometer for the handle height adjustment. The chain modification was adapted to their height. When the handle remained in a neutral position, without any external force being implemented, the examiner calibrated the machine to show a zero price. A demonstration of the test and an attempt without exerting much effort were performed. Then, the participant was asked to pull the handle as much as possible for 5 s, trying to maintain the attachment of his/her heels to the dynamometer for the entire time. To avoid the Valsalva technique, he/she was asked to exhale during the contraction. Three maximal efforts were performed and recorded in kilograms. A 30 s rest period between the trials was implemented. 

Moreover, the Concept2 DYNO isokinetic dynamometer (Concept2, Morrisville, VT, USA) was used to evaluate the participant’s lower limb strength during the leg press exercise. The participant commenced from a seated position, placing his/her feet on the leg points, and was instructed to practice three trials, without exerting much effort. For the subsequent three efforts, the participant was required to exert maximal strength. The breathing pattern suggested was exhaling during the pushing phase and inhaling during the pulling [54]. Upon completion of these attempts, the maximum leg strength and the average of the three trials were tallied in kilograms. 

### 2.7. 30 s Sit-to-Stand Test

The 30 s Sit-to-Stand test is a reliable, valid, and widely-used test that measures leg strength and exercise capacity in cardiac patients [55,56]. To conduct the assessment, the participant started from their sitting position with his/her back straight. The seat was 43.2 cm high, non-adjusted for each patient, and was located against the wall to prevent moving during the test [57,58]. Their feet were placed on the floor at shoulder width apart, and their arms were crossed in front of their chest [57]. After the test was demonstrated, the participant was asked to try one repetition, to become familiar with the exercise [57] and ensure its correct execution. For the test procedure, the participants were instructed to stand and sit repetitively, as rapidly as possible, for a duration of 30 s. For a repetition to be counted, the participant should be erected, full and straight, and return to their initial position [57]. Participants were instructed to maintain a normal breathing pattern throughout the test to prevent a potential Valsalva maneuver [55]. The number of repetitions a person completed was recorded. 

### 2.8. Activities-Specific Balance Confidence (ABC) Scale 

Thereafter, participants were asked to fill in the ABC Scale, which is a 16-item structured questionnaire, developed by Powell and Myers [59] to measure perceived balance confidence during the performance of daily activities. It is feasible to detect loss of balance confidence in seniors who exhibit higher levels of functioning. Each item is assessed from zero to 100%, with lower scores indicating lower levels of balance confidence. The total score is calculated by adding the score of each item and dividing it by 16. Scores above 50 and below 80 demonstrate a moderate degree of functioning [50].

### 2.9. Number of Falls

According to the World Health Organization (WHO) in 2021 [60], a fall is described as “*an event which results in a person coming to rest inadvertently on the ground or floor or other lower level*”. The participants were asked to rate the number of falls during the past year (over the past 12 months). Fallers were classified as those who experienced at least one fall over the previous year. 

### 2.10. Sample Size Estimation

The sample size calculation was based on hypothesized differences in static balance parameters between patients with HF and age-matched healthy individuals. Based on the results of an earlier study [42], we assumed that the Patients with HFpEF group would have impaired static balance compared to healthy controls. Using a two-tailed test of significance with a 0.05 two-sided significance level, to achieve a power of 80%, it was estimated that a total of 34 subjects per group were required. The goal was to recruit at least 27 subjects into each group, assuming a 20% dropout rate.

### 2.11. Statistical Analysis

Prior to statistical analysis, all measures were checked for violations of the normality of the distribution and homogeneity of variance assumptions using the Shapiro–Wilk and Levene’s tests, respectively. Between-group differences in height, weight, body mass index, gender, lean fat mass parameters, systolic blood and diastolic pressure, left ventricular (LV) indices, functional strength tests, falls, and balance confidence were determined, employing an independent samples *t*-test. Differences between the two groups and two balance tasks in the COP measures were analyzed using a two-way (2 × 2) ANOVA with repeated measures on the balance task. This test was chosen considering the Group as an independent factor, having two levels: a. Patients with HFpEF, and b. healthy subjects, and the Task as the dependent-repeated factor, having two levels: a. bipedal stance, and b. Tandem-Romberg stance. A post hoc Bonferroni test was used to examine pairwise comparisons between tasks when a significant Group-by-Task interaction was detected. A Pearson correlation coefficient analysis was performed to assess possible relationships among static balance, strength, mass indexes, balance confidence, and fall rate. Particularly, a partial correlation coefficient was chosen to describe the strength of a linear relationship between two variables, either between static balance and/or balance confidence and/or fall rates in the two groups, either between strength parameters, and/or lean fat mass, and/or ABC, and/or falls in Patients with HFpEF. Furthermore, a network analysis was executed to clarify (positive and negative) relations between the parameters. A Glasso package was used to compute a sparse gaussian graphical model with the graphical lasso, selecting as tuning parameter the Extended Bayesian Information criterium (EBIC). The level of statistical significance was determined as *p* < 0.05. The statistical analysis was performed using IBM Statistical Package for Social Sciences (IBM Corp. Released 2020. IBM SPSS Statistics for Windows, Version 29.0. Armonk, NY, USA: IBM Corp.)

## 3. Results

### 3.1. Participants’ Characteristics

Baseline characteristics of the patients and healthy controls are presented in Table 1. No significant differences were observed in age, height, weight, body mass index, gender, and blood pressure between the two groups (*p* > 0.05). Lean fat mass, lean fat mass%, and free fat mass index were significantly lower among Patients with HFpEF compared to healthy controls (*p* < 0.038, *p* < 0.001, and *p* < 0.005, respectively).

### 3.2. Functional Strength Tests

Functional strength parameters are presented in Table 2. In Patients with HFpEF, the number of repetitions performed on the 30 s chair stand test, the kilograms pushed on the Dyno strength test, and the hamstring strength executed, were lower by 54.2% (*p* < 0.001), 52.2% (*p* < 0.001), and 26.8% (*p* < 0.020), respectively, compared to healthy controls. 

### 3.3. Falls and Balance Confidence

Patients with HFpEF demonstrated a higher prevalence and number of falls within the past year, presenting with statistically significant differences compared to healthy controls (*p* < 0.01) (Table 3). ABC scores were statistically lower in Patients with HFpEF, by 21.5% (*p* < 0.01) compared to the healthy controls, indicating lower balance confidence. 

### 3.4. Static Balance Parameters 

**Figure 1:**
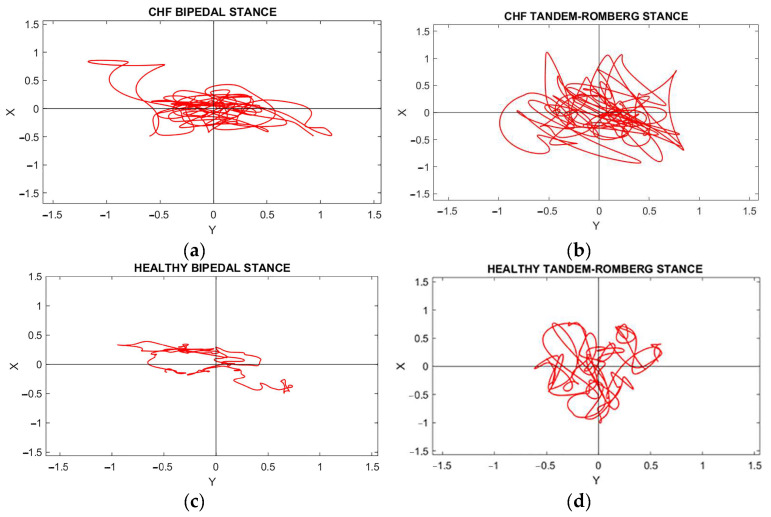
A Stabilogram, which depicts the trajectory of the COP displacement during balance maintenance. ML vs. AP COP plots of a patient with HFpEF and a healthy subject in bipedal Stance (**a**,**c**) and Tandem-Romberg Stance (**b**,**d**).

Representative ML vs. AP COP plots of a patient with HFpEF and a healthy subject performing the two static balance tasks are illustrated in Figure 1. A deeper look in Figure 1 demonstrates that the plots are amplified in patients with HFpEF in both tasks, showing the difference between the two groups.

#### 3.4.1. Range of the Center of Pressure Displacement (COP_RANGE_)

##### COP_RANGE_ in Mediolateral Axis

**Figure 2:**
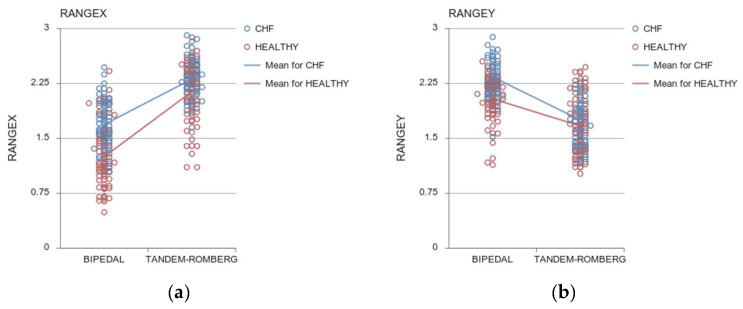
Range of COP in the ML (**a**) and AP (**b**) directions. Open circles indicate individual values for the HF (in blue) and healthy (red) participants. Lines represent the group means.

- Analyses revealed a main effect of Group in the Range of the COP displacement in the mediolateral axis (ML), (COP_RANGEX_: F (1, 142) = 53.796, *p* < 0.001, η_p_^2^ = 0.275) (Figure 2a) suggesting that Patients with HFpEF swayed more in the ML direction in comparison with healthy controls in both tasks. Specifically, in the bipedal stance, there was a 26% difference (*p* < 0.001) in the Range COP displacement in the ML direction between patients and healthy controls. Similarly, Patients with HFpEF revealed increased sway in the Tandem-Romberg stance by 8.45% (*p* < 0.001).
- A main effect of the task was noted, indicating that as the level of difficulty increased, the Range of COP sway in the mediolateral axis also increased (COP_RANGEX_: F (1, 142) = 322.779, *p* < 0.001, η_p_^2^ = 0.694).
- A Task × Group interaction showed that the difference between the two groups was larger in the bipedal than in the Tandem-Romberg stance (COP_RANGEX_: F (1, 142) = 9.216, *p* = 0.003, η_p_^2^ = 0.061). Post hoc analysis revealed larger COP sway in Patients with HFpEF than in controls in the bipedal stance (*p* < 0.001). In the Tandem-Romberg stance, even if the difference between the two groups was smaller, the difference was still significant (*p* < 0.001). In particular, the COP Range difference in the ML direction between the two tasks was augmented by 27.27% (*p* < 0.001) in Patients with HFpEF and by 41.31% (*p* < 0.001) in healthy controls.

##### COP_RANGE_ in Anteroposterior Axis

- A main effect of Group in the Range of COP displacement in the anteroposterior (AP) direction (COP_RANGEY_: F (1, 142) = 26.783, *p* < 0.001, η_p_^2^ = 0.106) revealed that Patients with HFpEF swayed more in the AP direction compared to healthy participants (group difference in bipedal by 12.45% and in the Tandem-Romberg stance by 4.95%, respectively, Figure 2b).
- Concerning the Task, both groups decreased the COP Range in the AP axis from the bipedal to the Tandem-Romberg stance (COP_RANGEY_: F (1, 142) = 26.783, *p* < 0.001, η_p_^2^ = 0.106).
- The statistically significant Task x Group interaction suggests that the difference between the two groups was larger in the bipedal in comparison with the Tandem-Romberg stance (COP_RANGEY_: F (1, 142) = 6.424, *p* = 0.012, η_p_^2^ = 0.043).

#### 3.4.2. Standard Deviation of the Center of Pressure Displacement (COP_SD_)

##### COP_SD_ in Mediolateral Axis

**Figure 3:**
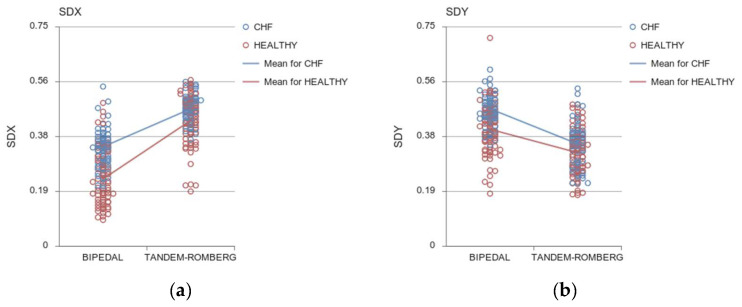
SD of COP in the ML (**a**) and AP axes (**b**). Open circles indicate individual values for the HF (in blue) and healthy (red) participants. Lines represent the group means.

- A main effect of the Group on the Standard Deviation (SD) of COP displacement in the ML direction (COP_SDX_: F (1, 142) = 57.845, *p* < 0.001, η_p_^2^ = 0.289), suggested that Patients with HFpEF deviated more than healthy controls in both tasks in the ML direction (between-group difference 30.3% in the bipedal and 8.48% in the Tandem-Romberg stance) (Figure 3a).
- Both groups increased their Standard Deviation of the COP displacement from bipedal to Tandem-Romberg stance (COP_SDX_: F (1, 142) = 286.223, *p <* 0.001, η_p_^2^ = 0.668).
- A Task × Group significant interaction (COP_SDX_: F (1, 142) = 12.648, *p* < 0.001, η_p_^2^ = 0.082) indicated that Patients with HFpEF and healthy participants differed more in the bipedal stance rather than in the Tandem-Romberg stance. The difference between the bipedal stance and the Tandem-Romberg stance was amplified in Patients with HFpEF by 28.28% and in healthy subjects by 46.5%, respectively (*p* < 0.001).

##### COP_SD_ in Anteroposterior Axis

- In Patients with HFpEF, SD of COP displacement in the AP direction exceeded the SD of healthy participants (COP_SDY_: F (1, 142) = 28.430, *p* < 0.001, η_p_^2^ = 0.167), revealing higher instability of Patients with HFpEF in the bipedal stance (Figure 3b).
- A statistically significant main effect of the Task was detected, highlighting that in the bipedal stance, both groups were more variable in the AP direction than in the Tandem-Romberg stance (COP_SDY_: F (1, 142) = 125. 805, *p* < 0.001, η_p_^2^ = 0.421).
- A significant Task × Group interaction (COP_SDY_: F (1, 142) = 5.888, *p* = 0.017, η_p_^2^ = 0.040) confirmed a greater difference in the bipedal stance than the Tandem-Romberg stance. Particularly, in Patients with HFpEF, COP SD sway in the AP direction decreased by 28.26% in Tandem-Romberg, where the two groups differed by 17.94% (*p* < 0.001).

### 3.5. Network Analysis

**Figure 4:**
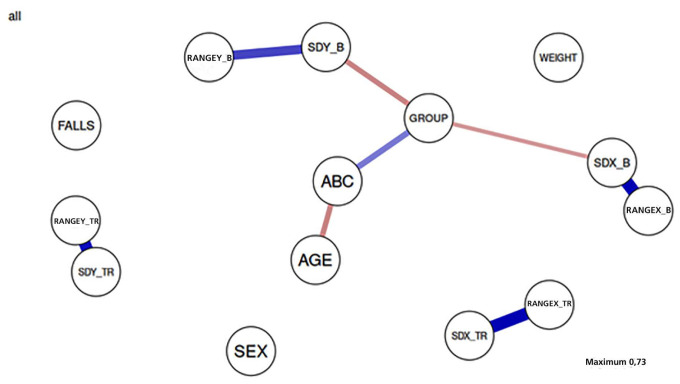
Network analysis between parameters.

According to a network analysis, presented in Figure 4, there were highlighted positive and negative relationships. The network revealed strong positive associations between the two balance indices in the bipedal and TR stances, while these were moderately linked to the ABC, Group, and age factors.

### 3.6. Correlations

#### 3.6.1. Correlations between Falls, ABC, and Balance Parameters

**Figure 5:**
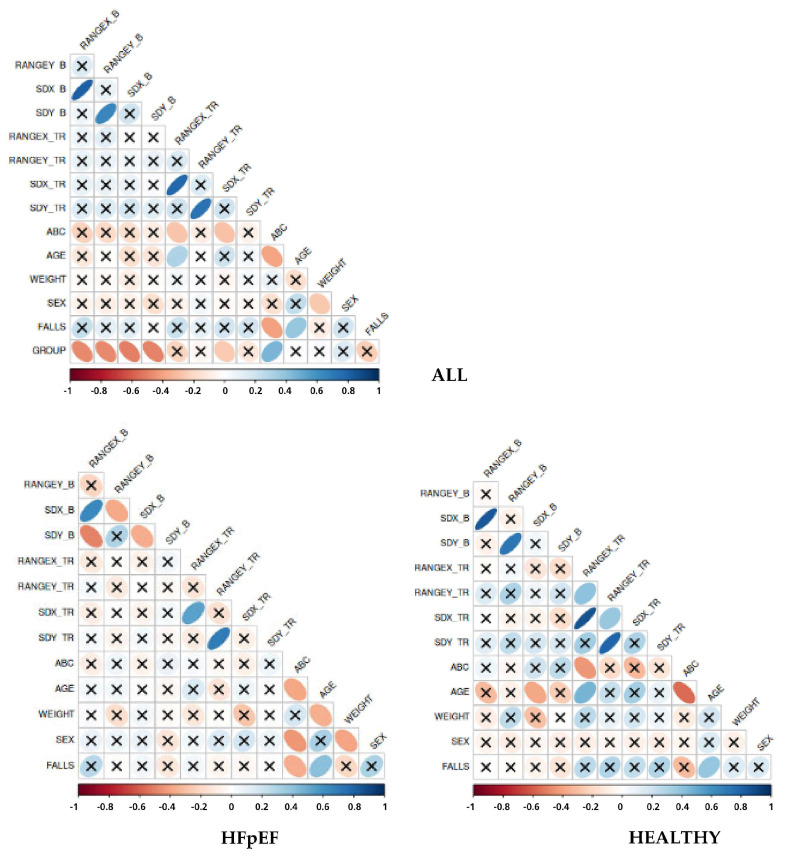
Associations between ABC, falls, patients’ characteristics, and static balance parameters.

Correlation analysis was performed on ALL participants and separately for the two groups (Figure 5). Regarding all the participants, the results demonstrated that falls were negatively related to ABC and positively related to age. Furthermore, Group was negatively associated with the Range and SD COP parameters suggesting static balance as a discriminant of HF pathology. Sex appeared to be slightly adversely correlated with weight, whereas age presented a negative correlation with the ABC scale.

Considering differences within Group, in Patients with HFpEF, sex seemed to be negatively related to weight and ABC scale whereas the correlation was not present in the healthy population. Moreover, in Patients with HFpEF, an adverse relationship between weight and age was depicted. Regarding the other parameters, there were no statistically significant correlations.

#### 3.6.2. Correlations between Strength Parameters, Lean Fat Mass, and ABC and Falls in Patients with HFpEF

**Figure 6:**
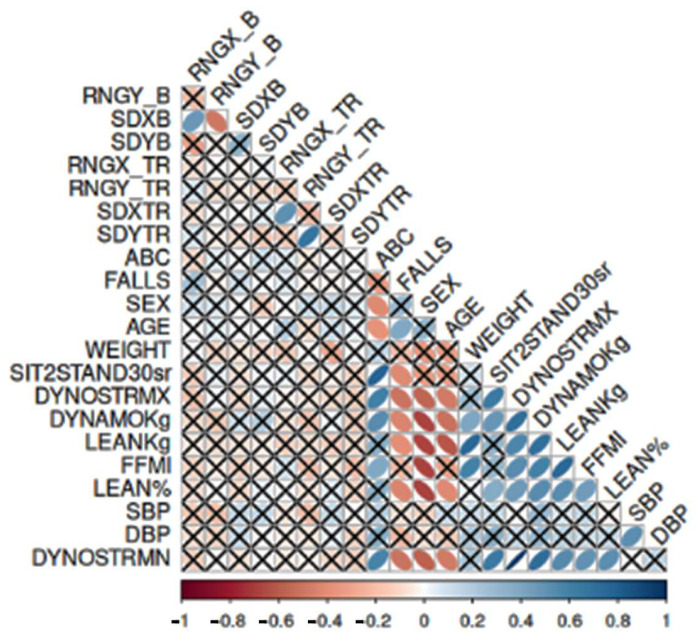
Strength, lean fat mass, falls, and ABC in Patients with HFpEF.

As shown in Figure 6, in Patients with HFpEF, there were correlations between strength parameters, lean fat mass, ABC, and falls, as well as between strength, FFMI, and ABC. An adverse correlation between falls and strength parameters was presented, as well as a strong negative relationship between Dyno strength test, Baseline strength dynamometer, lean fat mass, FFMI, and lean% with sex. Age was negatively correlated with Dyno strength test, Baseline strength dynamometer and lean fat mass, whereas ABC scale was positively associated with Sit-to-Stand Test, Dyno strength test, and Baseline strength dynamometer.

## 4. Discussion

The results of the present study revealed impaired static balance performance in Patients with HFpEF that was more pronounced in the bipedal stance than in the TR stance. Interestingly, laboratory-assessed static balance metrics showed little to no correlation with lean muscle mass, balance confidence, falls, or strength. On the other hand, age, muscle strength, lean fat mass, and balance confidence were related to the rate of falls.

Specifically, our Patients with HFpEF demonstrated worse static balance than healthy controls. Our findings are in line with Tanriverdi et al. [42] who noted that individuals with HF show greater balance deficits compared to healthy individuals, attributing the result to reduced peripheral quadricep muscle strength of their patients. On the other hand, it is known that static balance performance greatly depends on the activation of more distal muscles surrounding the ankle joint [61]. To further explore this hypothesis about the role of peripheral muscle strength in static balance, we tested our participants in two different balance tasks of increasing difficulty. We expected that the between-group differences would be enlarged in the more demanding balance task of the Tandem-Romberg stance, which challenges balance in the mediolateral direction [62,63] and requires the development of a counter-rotation (antigravity) torque by the hip abductors/adductors. Contrary to our expectations, the between-group differences were decreased in the TR stance. This finding suggests that Patients with HFpEF possibly maintained comparable levels of hip abductor/adductor strength, while related differences due to HF may be more prominent in the bipedal stance that is controlled by the ankle dorsi/plantar flexors [61].

Our results showed that the bipedal stance was a better descriptor of the Group difference. Between-group differences tended to decrease when balance was challenged. Previous studies, comparing the bipedal stance and the Tandem-Romberg stance among older adults, claimed that in the bipedal stance, COP displacement was eliminated in the ML axis, whereas in the Tandem-Romberg stance individuals were more stable in the AP axis [64]. Moreover, it was found that augmenting the task difficulty in healthy older adults was related to reduced COP variability in the AP axis and inclined COP complexity in the ML axis [65]. The Center of Pressure distribution mechanism is responsible for controlling anteroposterior movements for the bipedal position while also securing mediolateral sway in the Tandem stance [66]. Moreover, it was discovered that both feet were engaged in Tandem-Romberg control, suggesting that the front foot is related to balance in the anteroposterior axis and the rear foot in the mediolateral axis [67]. Different strategies and muscle groups were also associated with the bipedal stance and the Tandem-Romberg stance. Specifically, anteroposterior sway in the bipedal stance is controlled by the ankle strategy, whereas mediolateral sway is secured by hip load/unload strategy [68]. Respectively, in the Tandem-Romberg stance the ankle strategy is related to anteroposterior axis control, while the hip/load strategy controls stability in the mediolateral axis [66]. With advancing age, narrowing the base of support was related to greater hip muscle involvement, which may be explained by the greater motor unit loss in distal compared to proximal muscles, since the incidence of peripheral mechanical trauma is higher in the nerves feeding distal muscles [61,69,70]. Plantar flexor muscles of Patients with HF were found to have higher fatiguability rates by 54%, faster time to peak tension, and half relaxation time, whereas knee extensor muscles were reported to reach fatigue more easily, by 45%, and have a remarkably faster relaxation time [71].

The results of our study indicated that static balance assessed in controlled lab conditions had little if no relationship to falls, strength, lean muscle mass, and balance confidence. It is already known that static balance is mostly linked to postural control in standing positions [72]. On the other hand, more falls occurred during incorrect weight shifting, followed by trip or stumble, hit or bump, loss of support, and collapse, whereas slipping concerned only 3%. Respectively, the most prevalent activities related to the incidence of falls were forward walking, standing quietly, and sitting down [73]. Considering patients with HF, apart from the clinical characteristics of the patient and the frailty and sarcopenia risk factors, increased fall rates may occur due to medications, environmental factors, and cognitive impairment [27].

Our results followed Hu et al. (2024), suggesting that none of the static balance Tandem-Romberg parameters are related to falls in older adults [24]. The researchers accredit the non-significant relationship to the relatively healthy participants having no falls during the study [24]. Previous studies claimed that among older adults, greater ML COP displacement is related to a three times greater fall risk [74,75], whereas others suggested COP in the AP axis was a discriminative factor, linked to the chance of suffering a major injury after a fall incident [76,77]. The difference between the ML and AP axes as determining factors was probably related to the stratification parameters of the systematic review [77]. Nevertheless, to classify fallers and non-fallers in older adults, they considered the sway path to be the most accurate, specific, and sensitive parameter to determine the group differences [78]. Furthermore, among other studies, mean velocities were regarded as the factors used in distinguishing people with and without falls, presenting AP measurements as more discriminative [77].

Our results, with respect to this measure, revealed that Patients with HFpEF experienced a higher number of falls over the last 12 months. Patients with HF are more prone to falls by 43% in comparison with patients with other chronic diseases [35], for various reasons [27]. Impaired static balance, lower muscle strength, and a greater number of falls are among their characteristics [42]. Nevertheless, no other study correlated static balance to the number of falls in patients with HFpEF.

With static balance tasks, such as quiet standing, it is probable not to be associated with falls and muscle strength because they do not necessitate as much muscle activation [79] and may involve the activation of different cortical areas as compared to a dynamic process [80]. A recent study suggested that static balance in older adults demonstrated more delta activation in the anterior cortex while they recruited sensorimotor areas and presented higher muscle activity when performing a dynamic task [79]. During a static test with a visual oddball task, the participants expanded the activation over the sensorimotor and occipital cortices, to preserve postural control [79]. Considering, the dynamic nature of a fall, it may explain why the evaluation of static balance in controlled lab conditions had little if no relationship to falls, and strength parameters.

Our findings are in parallel to previous studies reporting that laboratory static balance parameters are not correlated to muscle strength [81,82]. Song et al. [82] assessed COP root mean square in older adults, as an indicator of static postural control, measuring 30 s bipedal stance with feet together. Ankle dorsi/plantar flexion and hip abduction were evaluated as indexes related to static balance control [82,83]. A weak-to-moderate association between proprioception and static balance, and a weak link between cutaneous sensitivity and static balance control were reported [82]. On the contrary, in frail elderly individuals who were highly susceptible to falls, the strength of the antigravity muscle facilitates improved postural balance [84]. Correlations between one-leg stance test in computerized balance platform, knee extensor, and ankle dorsiflexion muscle strength found in kidney transplant recipients [85], considering that the postural stability during backward motions is related to ankle dorsiflexor muscles [85,86].

We found that lean muscle mass had no relationship with COP parameters. In elderly outpatients, no associations were presented between COP movement and muscle properties [87]. Authors attributed their results to the heterogeneity among elderly outpatients, the existence of several comorbidities, and the decline of several systems linked to standing balance, highlighting that a decline in neural or sensory patients’ systems may be related to increased muscle mass or strength as a compensatory strategy [87]. Furthermore, in line with our study, muscle characteristics did not exhibit any relations with oscillations in the center of gravity measured in the bipedal stance with feet together for one minute, in older patients. The researchers suggested that static balance tests that do not involve muscle contraction should not be utilized to evaluate poor muscle features [88]. Nevertheless, sarcopenia defined by appendicular skeletal muscle mass, handgrip strength, and gait speed, was shown to be related to ML COP excursion range in Tandem-Romberg in older adults [24].

Our results showed no significant relationship between static balance parameters and balance confidence. A negative relationship between ML excursion range, short axis range, and sway area in CoM and fear of falling was found by Hu et al. [24] during Tandem-Romberg, claiming that healthy older adults may exhibit better postural control and balance confidence, considering that balance confidence is higher with eyes open. Additionally, no relationship was found between COP parameters and ABC scores in elderly women with knee osteoarthritis, relating the result to other conditions, such as self-efficacy, anxiety, or depression, that were not assessed [89].

Fall rates in patients with HF and advancing age were increased, a finding which is in line with Denfeld et al. [28]. In adults aged over 50 years, cardiovascular disease and the prevalence of falls were positively correlated [90]. Cardiovascular pathology includes a wide variety of disorders that can increase the risk of falls, especially in older people [91].

According to our results, the muscle strength deterioration was related to a greater number of falls, presenting an increased correlation with maximal strength in Dyno Concept2. The risk of falling was significantly decreased for each kilogram of muscle mass or strength gained [92]. Low lower limb strength and obesity, defined as dynapenic obesity, was considered to be a predictive factor of higher fall prevalence in middle-aged and older adults than sarcopenic obesity [93].

Balance confidence was found to exhibit a negative relationship with the number of falls. This result is aligned with previous studies, presenting similar ABC scores and fall rate with age-matched patients with HF with augmented and not augmented fall risk [94]. Nevertheless, the difference of the studies lies in the fact the number of falls was recorded during the last 3 months, and 37.5% of the patients were patients with HF and preserved ejection fraction. The reduced number of falls in our study may be related to the preserved ejection fraction.

We acknowledge that in our study there are some limitations. Regarding static balance parameters, we evaluated solely Range and Standard Deviation COP parameters considering them as discriminative factors between the two groups. Secondly, we assessed Tandem-Romberg’s COP displacement, using the right foot in front, for all the participants. However, limb dominance was not found to play a significant role in Tandem stance control in healthy adults [52]. Moreover, we evaluated the muscle strength of the lower limbs and not of specific muscle groups. Muscle strength of specific muscle groups of the lower extremities may be related to static balance in Patients with HPpEF. Further research is required in the future regarding balance in HFpEF, falls, and the correlated factors. In particular, the exploration of the potential moderating effects of comorbidities was not examined. Although they could provide a more nuanced understanding of the factors influencing balance and fall risk in HFpEF patients. Future studies involving a larger number of participants would be valuable for detecting significant differences or correlations between the groups, avoiding implications of the study’s power and generalizability, and verifying or exploring further our findings. Despite limitations in study design, our primary findings have apparent clinical implications.

## 5. Conclusions

The results of the present study indicated that Patients with HFpEF demonstrated lower static balance compared to healthy age-matched individuals. Moreover, as the lean muscle mass and the muscle strength were reduced, the number of falls increased in our patients. It is of high importance that bipedal stance predictability of impairment related to HF pathology was found to be superior to that of the more challenging Tandem-Romberg task. Our findings highlight the significance and need for a thorough assessment of balance and functional capacity using both laboratory and field-based measures in Patients with HFpEF, to achieve better clinical outcomes. Implementing appropriate balance and strengthening exercises, accordingly, in the cardiac rehabilitation programs, is highly recommended.

## Figures and Tables

**Table 1:** Participants’ characteristics.

Characteristics	Patients with HFpEF (*n* = 72) Mean ± SD	Healthy Controls (*n* = 72) Mean ± SD	*p*-Value
Age (years)	66.0 ± 11.6	65.3 ± 9.5	NS
Height (cm)	167.2 ± 10.7	168.1 ± 12.3	NS
Weight (kg)	83.4 ± 16.3	84.1 ± 10.0	NS
Body Mass Index (kg/cm^2^)	29.7 ± 4.5	28.4 ± 5.1	NS
Gender (female %)	45.8	58.3	NS
Lean Fat Mass (%)	63.2 ± 8.4	70.5 ± 9.3	<0.001
Lean Fat Mass (kg)	52.0 ± 12.1	57.3 ± 11.4	0.038
Free Fat Mass Index (kg/cm^2^)	18.0 ± 2.3	19.4 ± 1.8	0.005
Systolic Blood Pressure (mmHg)	124.5 ± 12.9	123.7 ± 6.2	NS
Diastolic Blood Pressure (mmHg)	71.7 ± 8.6	76.4 ± 8.2	NS
New York Heart Association Class (n)			
I	26.40%	-	-
II	62.50%	-	-
III	11.10%	-	-
Left Ventricular (LV) Indices			
LV Ejection Fraction (%)	56.0 ± 4.2	61.0 ± 5.3	<0.001
Ε/A ratio	2.1 ± 0.3	1.1 ± 0.8	<0.001
Ε/Ε′ av ratio	17.0 ± 3.1	7.4 ± 2.9	<0.001
Ε′ av (m/s)	0.07 ± 0.02	0.12 ± 0.03	<0.001
Medication			
Beta-blockers	58.60%	-	-
Anticoagulants/antiplatelet	78.60%	-	-
Statins	55.70%	-	-
ACE inhibitors/ARBs	54.30%	-	-
Diuretics	24.30%	-	-

The results are expressed as mean ± SD or n (%). NS—not significant; E—early diastolic peak flow velocity; A—late diastolic peak flow velocity; E′—peak early diastolic mitral annular velocity; ACE—angiotensin converting enzyme; ARBs—angiotensin receptor blockers.

**Table 2:** Functional strength tests.

Functional Strength Tests	Patients with HFpEF (n = 72) Mean ± SD	Healthy Controls (n = 72) Mean ± SD	*p*-Value
30 s Chair Stand Test (rep)	11.9 ± 3.6	18.4 ± 3.1	<0.001
Dyno Strength Test-Leg Press (kg)	47.3 ± 23.0	72.0 ± 36.8	<0.001
Baseline Leg Dynamometer (kg)	61.5 ± 31.8	78.0 ± 35.0	0.020

**Table 3:** Falls and balance confidence.

	Patients with HFpEF (n = 72) Mean ± SD	Healthy Controls (n = 72) Mean ± SD	*p*-Value
**Number of falls**	0.96 ± 1.28	0.26 ± 0.44	<0.01
% Fallers	41.7%	31.9%	<0.001
**Balance Confidence**			
ABC Scale Score	71.0 ± 16.9	86.3 ± 11.2	<0.01

## Data Availability

The datasets generated or analyzed during the current study are available from the corresponding author upon reasonable request.

## List of Abbreviations

AP	Anteroposterior Axis
BMI	Body Mass Index
BS	Bilateral Stance
COP	Center of Pressure
COPX	Center of Pressure X-Mediolateral Axis
COPY	Center of Pressure Y-Anteroposterior Axis
HF	Heart Failure
LVEF	Left Ventricular Ejection Fraction
ML	Mediolateral Axis
HFpEF	Heart Failure with Preserved Ejection Fraction
SD	Standard Deviation
TRS	Tandem-Romberg Stance
